# Effectiveness comparison of pembrolizumab versus other immune checkpoint inhibitors in neoadjuvant chemoimmunotherapy for esophageal squamous cell carcinoma

**DOI:** 10.3389/fcell.2026.1743881

**Published:** 2026-04-01

**Authors:** Mingduan Chen, Shuhan Xie, Zhinuan Hong, Mingqiang Kang

**Affiliations:** 1 Department of Thoracic Surgery, Fujian Medical University Union Hospital, Fuzhou, China; 2 Key Laboratory of Cardio-Thoracic Surgery (Fujian Medical University), Fujian Province University, Fuzhou, China; 3 Key Laboratory of Ministry of Education for Gastrointestinal Cancer, Fujian Medical University, Fuzhou, China; 4 Clinical Research Center for Thoracic Tumors of Fujian Province, Fuzhou, China; 5 Key Laboratory of Gastrointestinal Cancer (Fujian Medical University), Ministry of Education, Fuzhou, China

**Keywords:** domestic immune drugs, imported immune drugs, landmark analysis, neoadjuvant chemoimmunotherapy, survival analysis

## Abstract

**Background and purpose:**

Neoadjuvant chemoimmunotherapy (nICT) has emerged as a promising strategy for esophageal squamous cell carcinoma (ESCC). However, the efficacy and safety comparison of imported and domestic immune drugs remain unclear. This study aimed to compare short- and long-term efficacy between domestic and imported immunotherapeutic agents.

**Method:**

A total of 128 ESCC patients were retrospectively analyzed. Domestic immune drugs included sintilimab, tislelizumab, camrelizumab, and toripalimab, while the imported drug was pembrolizumab. Propensity score matching (PSM) was applied to balance baseline characteristics. Cox analysis, Kaplan-Meier survival and landmark analysis were used for survival analysis. Study outcomes included recurrence-free survival (RFS), locoregional recurrence-free survival (L-RFS), distant metastasis-free survival (D-MFS), pathological response, and postoperative complications.

**Result:**

97 patients received domestic drugs and 31 received imported drugs. No significant differences were observed in RFS (p = 0.351), L-RFS (p = 0.075) and D-MFS (p = 0.772) before PSM. After PSM, survival outcomes remained comparable. 12th-month landmark analysis also showed no significant differences between two categories. Although imported drug was associated with a higher MPR rate before matching (p = 0.005), this difference was not significant after PSM (p = 0.128). Complication rates were similar except for fewer pleural effusions (p = 0.026) and more electrolyte disturbances (p = 0.030) in the imported group after PSM.

**Conclusion:**

Domestic and imported immune drugs demonstrated comparable long-term survival and safety in ESCC patients receiving nICT. Although imported agents may offer a modest advantage in pathological response, both categories remain effective options for clinical use.

## 1 Introduction

Esophageal cancer (EC) was ranked as the sixth leading cause of cancer-related mortality worldwide (Cao et al., 2021). In China, more than 50% of patients with esophageal squamous cell carcinoma (ESCC) are diagnosed at a locally advanced stage upon initial presentation, requiring neoadjuvant therapy (Xing et al., 2021). Neoadjuvant chemoradiotherapy (nCRT) and neoadjuvant chemotherapy (Babic et al., 2020; Hashimoto et al., 2020) remain the primary standard treatment options for locally advanced ESCC. However, as growing evidence supports the effectiveness and safety of immunotherapy, neoadjuvant chemoimmunotherapy (nICT) combined with subsequent surgical resection is emerging as a novel and highly promising strategy.

The phase II Keystone-001 trial provided strong clinical evidence for the safety and efficacy of pembrolizumab, and preliminarily verified the feasibility and potential advantages of pembrolizumab with chemotherapy as a treatment option for resectable ESCC (Shang et al., 2024). In the another phase II clinical trial, the combination of sintilimab and chemotherapy as a neoadjuvant regimen demonstrated favorable safety and efficacy (Guo J. et al., 2025). Then, the real-world study by Cai further confirmed the survival benefit of sintilimab (Cai et al., 2024). Similarly, the efficacy and safety of other immunotherapeutic agents including camrelizumab and toripalimab were proved (Li et al., 2025; Zheng et al., 2024; Liu et al., 2025; Gao et al., 2022). However, although these immunotherapeutic agents could confer survival benefits to the majority of EC patients, the selection of an optimal immunotherapeutic agent remains a clinical challenge for both clinicians and patients from the perspectives of clinical efficacy, safety and medical cost.

Given the wide range of immunotherapy regimens available for EC and the absence of direct comparative studies evaluating their efficacy, this study aims to systematically compare imported and domestic immunotherapeutic agents across three key perspective: postoperative complications, pathological response, and long-term prognosis, providing clinicians and patients with evidence to inform treatment decision-making.

## 2 Methods

### 2.1 Study population

Esophageal cancer patients from our medical center were enrolled between 1 January 2019, and 30 September 2022. The inclusion criteria were (1) histologically confirmed ESCC at clinical stages II-IVa (2) receipt of at least one cycle of neoadjuvant chemoimmunotherapy; and (3) receipt of R_0_ esophagectomy. The exclusion criteria included (1) incomplete clinical data (2) the presence of other primary malignant tumors (3) receipt of other neoadjuvant therapy (including nCT or nCRT); and (4) undergoing salvage or palliative surgery.

### 2.2 Treatment protocol

The pre-treatment assessment and clinical staging were conducted utilizing gastroscopy, contrast-enhanced computed tomography scans and color Doppler ultrasound. Additionally, positron emission tomography-computed tomography was performed when deemed clinically necessary.

The chemotherapy regimen generally consisted of a tri-weekly administration of a combination of nab-paclitaxel/docetaxel and platinum-based agents. The immune drugs included sintilimab, tislelizumab, camrelizumab, toripalimab, pembrolizumab, administered at a dose of 200/240 mg tri-weekly. In this study, the immunotherapeutic agents of sintilimab, tislelizumab, camrelizumab and toripalimab were categorized as domestically produced drugs (China), whereas the pembrolizumab was classified as an imported drug (Xu et al., 2025).

The treatment efficacy was evaluated 3 to 4 weeks following the completion of either two or three cycles of neoadjuvant therapy. In instances where surgical intervention was deemed appropriate, patients subsequently underwent minimally invasive esophagectomy. A standard two-field lymph node dissection was routinely conducted, while the three-field lymph node dissection was reserved for cases where cervical lymph node metastasis was suspected.

Typically, adjuvant therapy (AT) was recommended for ESCC patients staged as ypT_0-4a_N_+_M_0_. In contrast, for those with ypT_0-4a_N_0_M_0_ disease, active surveillance was considered a viable alternative, particularly in individuals achieving pathological complete response (pCR). Additionally, the decision to administer AT was determined through a multidisciplinary evaluation that incorporated pathological findings, overall health status, and patient-specific treatment preferences (Xie et al., 2025a).

### 2.3 Relevant definition and follow-up

The primary outcome was recurrence-free survival (RFS), which was measured as the duration from the date of surgery to the occurrence of any recurrence, including locoregional recurrence and/or distant metastasis. The secondary outcomes included locoregional recurrence-free survival (L-RFS), defined as the time until locoregional recurrence, and distant metastasis-free survival (D-MFS), defined as the time to the development of distant metastasis (Hong et al., 2025).

A standardized follow-up protocol was recommended. During the first 2 years post-surgery, patients underwent routine assessments every 3–6 months. From the third to the fifth year, evaluations were scheduled semiannually, followed by annual follow-ups thereafter. Regular examinations for follow-up included tumor markers, chest CT and head MRI; if necessary, endoscopic examination or PET-CT can also be conducted. Locoregional recurrence and distant metastasis are usually determined based on imaging examination results; if feasible, pathological biopsy should be conducted for a definitive diagnosis. When necessary, multi-disciplinary consultation will also be organized.

### 2.4 Statistical analysis

Continuous variables are presented as mean ± standard deviation and compared using the Mann-Whitney U test or Student’s t-test; categorical variables are shown as frequency (proportion) and analyzed with the chi-square test or Fisher’s exact test. Propensity score matching (PSM) was performed by estimating propensity scores via logistic regression, followed by applying a nearest neighbor matching algorithm without replacement, using a caliper of 0.1 and a 1:1 matching ratio (Bruna et al., 2025). Matching variables included sex, age, smoking history, drinking history, BMI, ASA, tumor location, neoadjuvant chemotherapy regimes, lymph node dissection fields, and clinical stage. Survival differences were evaluated through Kaplan-Meier survival curves and 12-month landmark analysis. Univariate and multivariate Cox regression analysis were used to examine risk factors. Data analysis was performed using SPSS software (Version 27) and R software (Version 4.3.1), with statistical significance defined as p < 0.05.

## 3 Results

### 3.1 Patients characteristic

A total of 128 ESCC patients were enrolled in this study. Among them, 102 were male, and 61 were aged 60 years or younger. A total of 97 patients toke domestic immune drugs, while 31 chose imported one. Detailed baseline characteristics are presented in Table 1.

**TABLE 1:** Detailed baseline characteristics of ESCC patients.

Variables	Total (n = 128)
Sex	
Male	102 (79.69%)
Female	26 (20.31%)
Gender	
≤60	61 (47.66%)
>60	67 (52.34%)
Neoadjuvant chemotherapy regimen	
Nab-TP	100 (78.12%)
Others	28 (21.88%)
Immune drugs	
Domestic	97 (75.78%)
Imported	31 (24.22%)
Field	
2-Filed	114 (89.06%)
3-Filed	14 (10.94%)
BMI	
<18.5	13 (10.16%)
18.5–23.9	94 (73.44%)
≥24	21 (16.41%)
ASA	
2	114 (89.06%)
3	14 (10.94%)
Clinical stage	
II	46 (35.94%)
III	67 (52.34%)
IVa	15 (11.72%)
Smoking history	
No	51 (39.84%)
Yes	77 (60.16%)
Drinking history	
No	89 (69.53%)
Yes	39 (30.47%)
Tumor location	
Upper	13 (10.16%)
Middle	63 (49.22%)
Lower	52 (40.62%)
Yp stgae	
I	47 (36.72%)
II	16 (12.50%)
IIIa	26 (20.31%)
IIIb	37 (28.91%)
IVa	2 (1.56%)
pCR	
No	105 (82.03%)
Yes	23 (17.97%)
MPR	
No	69 (53.91%)
Yes	59 (46.09%)
Adjuvant therapy	
No	53 (41.41%)
Yes	75 (58.59%)

### 3.2 Cox analysis in original cohort

In the univariate Cox analysis, chemotherapy regimens, clinical stage, ypStage and MPR significantly affected RFS. Then, the multivariate Cox analysis showed only the ypStage and chemotherapy regimens was the significant independent risk factors. While, the imported immune drugs had no significant impact on survival (HR: 0.70; 95%CI: 0.32–1.50; P = 0.354), as illustrated in Table 2.

**TABLE 2:** The Univariate and Multivariate Cox analysis for ESCC after nICT.

Variables	Univariate cox analysis		Multivariate cox analysis	
	HR (95%CI)	P value	HR (95%CI)	P value
Sex				
Male	Reference	​	​	​
Female	0.58 (0.24–1.36)	0.209	​	​
Gender				
≤60	Reference	​	​	​
>60	1.33 (0.72–2.43)	0.362	​	​
Neoadjuvant chemotherapy regimen				
Nab-TP	Reference	​	Reference	​
Others	2.48 (1.32–4.65)	0.005	2.74 (1.44–5.22)	0.002
Immune drugs				
Domestic	Reference	​	​	​
Imported	0.70 (0.32–1.50)	0.354	​	​
Field				
2-Filed	Reference	​	​	​
3-Filed	1.05 (0.41–2.68)	0.913	​	​
BMI				
<18.5	Reference	​	​	​
18.5–23.9	1.16 (0.35–3.82)	0.81	​	​
≥24	3.31 (0.94–11.64)	0.062	​	​
ASA				
2	Reference	​	​	​
3	0.55 (0.17–1.78)	0.321	​	​
Clinical stage				
II	Reference	​	​	​
III	2.27 (1.10–4.68)	0.026	​	​
IVa	1.60 (0.55–4.70)	0.388	​	​
Smoking history				
No	Reference	​	​	​
Yes	1.32 (0.71–2.48)	0.38	​	​
Drinking history				
No	Reference	​	​	​
Yes	0.97 (0.51–1.86)	0.926	​	​
Tumor location				
Upper	Reference	​	​	​
Middle	1.43 (0.43–4.79)	0.563	​	​
Lower	1.72 (0.51–5.81)	0.383	​	​
ypStgae				
I	Reference	​	Reference	​
II	1.18 (0.31–4.46)	0.804	1.28 (0.34–4.83)	0.717
IIIa	1.73 (0.63–4.77)	0.291	1.77 (0.64–4.88)	0.271
IIIb	5.41 (2.41–12.10)	<0.001	5.82 (2.58–13.13)	<0.001
IVa	2.78 (0.35–22.24)	0.336	3.73 (0.46–30.40)	0.218
MPR				
No	Reference	​	​	​
Yes	0.33 (0.17–0.66)	0.002	​	​
Adjuvant therapy				
No	Reference	​	​	​
Yes	0.87 (0.48–1.60)	0.662	​	​

### 3.3 Survival analysis between two categories before and after PSM

**FIGURE 1:**
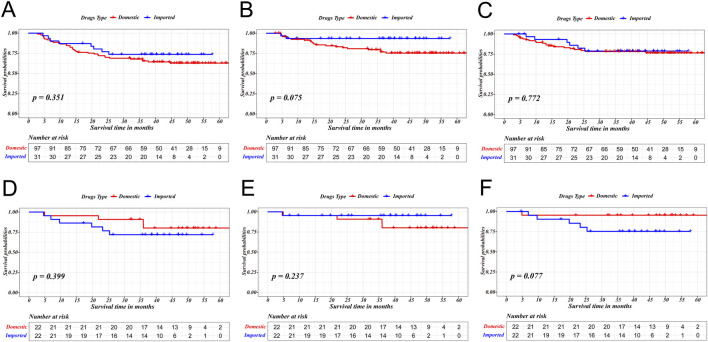
The survival difference between receipt of domestic drugs and imported drugs in terms of RFS **(A,D)**, L‐RFS **(B,E)** and D‐MFS **(C,F)** before and after PSM.

PSM was used to reduce confounding bias between domestic immune drugs and imported immune drugs, as shown in Table 3. Before matching, receipts of domestic drugs had similar RFS (p = 0.351), L-RFS (p = 0.075), and D-MFS (p = 0.772) compared to receipt of imported drugs. After PSM, substantial survival benefit were still exhibited between two group in RFS (p = 0.399), L-RFS (p = 0.237), and D-MFS (p = 0.077), as is shown in Figure 1.

**TABLE 3:** Comparison of domestic immune drugs and imported immune drugs before and after PSM.

Variables	Before PSM			After PSM		
	Domestic	Imported	P-value	Domestic	Imported	P-value
Sex	​	​	0.718	​	​	0.728
Male	78 (80.41%)	24 (77.42%)	​	17 (77.27%)	16 (72.73%)	​
Female	19 (19.59%)	7 (22.58%)	​	5 (22.73%)	6 (27.27%)	​
Gender	​	​	0.925	​	​	1.000
≤60	46 (47.42%)	15 (48.39%)	​	11 (50.00%)	11 (50.00%)	​
>60	51 (52.58%)	16 (51.61%)	​	11 (50.00%)	11 (50.00%)	​
Neoadjuvant chemotherapy regimen	​	​	0.059	​	​	0.6
Nab-TP	72 (74.23%)	28 (90.32%)	​	19 (86.36%)	21 (95.45%)	​
Others	25 (25.77%)	3 (9.68%)	​	3 (13.64%)	1 (4.55%)	​
Field	​	​	0.942	​	​	1.000
2-Filed	87 (89.69%)	27 (87.10%)	​	20 (90.91%)	21 (95.45%)	​
3-Filed	10 (10.31%)	4 (12.90%)	​	2 (9.09%)	1 (4.55%)	​
BMI	​	​	0.277	​	​	0.496
<18.5	8 (8.25%)	5 (16.13%)	​	2 (9.09%)	3 (13.64%)	​
18.5–23.9	71 (73.20%)	23 (74.19%)	​	20 (90.91%)	17 (77.27%)	​
≥24	18 (18.56%)	3 (9.68%)	​	0 (0.00%)	2 (9.09%)	​
ASA	​	​	1.000	​	​	1.000
2	86 (88.66%)	28 (90.32%)	​	20 (90.91%)	20 (90.91%)	​
3	11 (11.34%)	3 (9.68%)	​	2 (9.09%)	2 (9.09%)	​
Clinical stage	​	​	0.973	​	​	0.338
II	35 (36.08%)	11 (35.48%)	​	11 (50.00%)	7 (31.82%)	​
III	51 (52.58%)	16 (51.61%)	​	8 (36.36%)	13 (59.09%)	​
IVa	11 (11.34%)	4 (12.90%)	​	3 (13.64%)	2 (9.09%)	​
Smoking history	​	​	0.005	​	​	0.763
No	32 (32.99%)	19 (61.29%)	​	11 (50.00%)	10 (45.45%)	​
Yes	65 (67.01%)	12 (38.71%)	​	11 (50.00%)	12 (54.55%)	​
Drinking history	​	​	0.517	​	​	0.531
No	66 (68.04%)	23 (74.19%)	​	13 (59.09%)	15 (68.18%)	​
Yes	31 (31.96%)	8 (25.81%)	​	9 (40.91%)	7 (31.82%)	​
Tumor location	​	​	0.193	​	​	1.000
Upper	12 (12.37%)	1 (3.23%)	​	1 (4.55%)	1 (4.55%)	​
Middle	49 (50.52%)	14 (45.16%)	​	11 (50.00%)	12 (54.55%)	​
Lower	36 (37.11%)	16 (51.61%)	​	10 (45.45%)	9 (40.91%)	​

**FIGURE 2:**
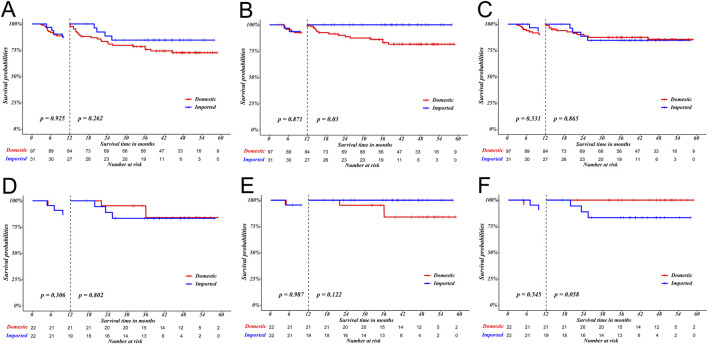
The 12-month landmark analysis between receipt of domestic drugs and imported drugs in terms of RFS **(A,D)**, L‐RFS **(B,E)** and D‐MFS **(C,F)** before and after PSM.

A landmark analysis was conducted using a 12-month cutoff. During 12-month period, similar survival outcomes between receipts of domestic drugs and receipts of imported drugs were observed in terms of RFS (p = 0.925), L-RFS (p = 0.871) and D-MFS (p = 0.531) in unmatched cohort. After PSM, no significant survival difference were observed in RFS (p = 0.306), LRFS (p = 0.987), and DMFS (p = 0.545). Similarly, beyond 12 months, no survival difference was observed in RFS (p = 0.802), L-RFS (p = 0.122) and D-MFS (p = 0.058) between two groups after PSM, as illustrated in Figure 2.

### 3.4 Comparison of pathological response and postoperative complications before and after PSM

Before PSM, imported drugs demonstrated superior efficacy in terms of MPR compared to domestic drugs, while exhibiting comparable rates of pCR. After PSM, no significant difference was observed between two groups in terms of MPR and pCR. However, a trend toward better MPR rate was observed in imported drugs, as is shown in Table 4.

**TABLE 4:** Comparison of pathological response between domestic immune drugs and imported immune drugs before and after PSM.

Variables	Before PSM			After PSM		
	Domestic	Imported	P-value	Domestic	Imported	P-value
pCR in tumor	​	​	0.030	​	​	0.220
No	73 (75.26%)	17 (54.84%)	​	15 (68.18%)	11 (50.00%)	​
Yes	24 (24.74%)	14 (45.16%)	​	7 (31.82%)	11 (50.00%)	​
pCR	​	​	0.192	​	​	0.728
No	82 (84.54%)	23 (74.19%)	​	17 (77.27%)	16 (72.73%)	​
Yes	15 (15.46%)	8 (25.81%)	​	5 (22.73%)	6 (27.27%)	​
MPR	​	​	0.005	​	​	0.128
No	59 (60.82%)	10 (32.26%)	​	12 (54.55%)	7 (31.82%)	​
Yes	38 (39.18%)	21 (67.74%)	​	10 (45.45%)	15 (68.18%)	​

In terms of pneumonia and anastomic leakage, two group had similar outcomes before and after PSM. While, imported drugs had a better control in pleural effusion (p = 0.026 after PSM) and worse performance in electrolyte disturbance (p = 0.003 after PSM), as is shown in Table 5.

**TABLE 5:** Comparison of postoperative complications between domestic immune drugs and imported immune drugs before and after PSM.

Variables	Before PSM			After PSM		
	Domestic	Imported	P-value	Domestic	Imported	P-value
Pneumonia	​	​	0.721	​	​	1.000
No	72 (74.23%)	24 (77.42%)	​	17 (77.27%)	17 (77.27%)	​
Yes	25 (25.77%)	7 (22.58%)	​	5 (22.73%)	5 (22.73%)	​
Anastomic leakage	​	​	1.000	​	​	0.469
No	86 (88.66%)	28 (90.32%)	​	22 (100.00%)	20 (90.91%)	​
Yes	11 (11.34%)	3 (9.68%)	​	0 (0.00%)	2 (9.09%)	​
Cardiac events	​	​	0.530	​	​	1.000
No	80 (82.47%)	24 (77.42%)	​	16 (72.73%)	16 (72.73%)	​
Yes	17 (17.53%)	7 (22.58%)	​	6 (27.27%)	6 (27.27%)	​
Chylothorax	​	​	0.145	​	​	0.469
No	96 (98.97%)	29 (93.55%)	​	22 (100.00%)	20 (90.91%)	​
Yes	1 (1.03%)	2 (6.45%)	​	0 (0.00%)	2 (9.09%)	​
Bleeding	​	​	0.964	​	​	1.000
No	93 (95.88%)	29 (93.55%)	​	21 (95.45%)	21 (95.45%)	​
Yes	4 (4.12%)	2 (6.45%)	​	1 (4.55%)	1 (4.55%)	​
Pleural effusion	​	​	0.014	​	​	0.026
No	58 (59.79%)	26 (83.87%)	​	11 (50.00%)	18 (81.82%)	​
Yes	39 (40.21%)	5 (16.13%)	​	11 (50.00%)	4 (18.18%)	​
Electrolyte disturbance	​	​	0.222	​	​	0.030
No	59 (60.82%)	15 (48.39%)	​	17 (77.27%)	10 (45.45%)	​
Yes	38 (39.18%)	16 (51.61%)	​	5 (22.73%)	12 (54.55%)	​

## 4 Discussion

Immunotherapy provides a novel treatment option for patients with locally advanced ESCC. Compared to these two traditional treatment modalities (nCRT and nCT), nICT enhanced long-term survival for ESCC. Besides, our previous study demonstrated that RFS associated with nICT was significantly superior to that observed with nCT, indicating that the great potential of nICT to be a standard treatment (Xie et al., 2025b). However, it remains unclear which type of immune drugs can bring better survival benefits to ESCC patients.

Postoperative complications associated with esophagectomy predominantly included postoperative pneumonia and anastomotic leakage, both of which are of significant concern to thoracic surgeons (Weber et al., 2025; van Hootegem et al., 2025). These complications result from both neoadjuvant therapy and surgery. In this study, no statistically significant differences were observed between domestic and imported pharmaceuticals concerning the two specified complications. Our previous multi-center research had proved that the additional immunotherapy did not enhance the rates of anastomotic leakage or postoperative pneumonia (Hong et al., 2023). This study provided additional confirmation that immune drugs from various manufacturers exhibited comparable risks of complications, thereby reinforcing the evidence supporting the safety of immune drugs for the treatment of ESCC.

pCR and MPR are two most common indicators for evaluating pathological response. While pCR is traditionally used as a prognostic surrogate in clinical trials (Sugumar et al., 2025), the study by Gou suggested MPR was a more appropriate surrogate for ESCC patients after neoadjuvant therapy (Guo X. et al., 2025). In this study, pCR rates were similar between two groups before and after PSM. In contrast, although no significant difference was observed in terms of MPR rate between the imported and domestic drugs after PSM, imported drugs still exhibited a higher MPR, implying imported immune drugs may offer better survival benefits. However, there is no statistical difference between the two groups in RFS, L-RFS, and D-MFS. For PD-1 drugs, whether it is imported pembrolizumab or domestic sintilimab, camrelizumab, etc., the target of these drugs is to block the binding of PD-L1 with PD-1 (Khoshghamat et al., 2021; Roberts et al., 2021). However, the subtle differences in antibody affinity and Fc segment modification may lead to slightly better blocking thoroughness and T cell protection in imported immune drugs, as well as a slight advantage in reversing deep T cell exhaustion, promoting memory T cell generation, and maintaining the stability of the immune synapse, thus showing potential survival advantages. However, prospective, large-scale head-to-head comparative studies are warranted to further clarify potential survival differences.

Previous research has demonstrated that the median and mean recurrence times for EC patients undergoing nICT are approximately 1 year following surgery (Yang et al., 2024). Therefore, a landmark analysis were performed at postoperative 12th month, showing no significant survival differences between two groups in RFS, L-RFS, and D-MFS within and beyond the postoperative 12 months, further confirming the comparable recurrence-free survival between two groups. Overall, although imported drugs may have certain advantages in MPR, the two are statistically comparable in terms of postoperative complications, pathological response and long-term prognosis, providing effective support and evidence for clinical decision-making.

To our knowledge, this is the first study to directly compare the short-term and long-term efficacy of domestic and imported immune drugs. However, this study is subject to several limitations. Firstly, the retrospective design inherently introduces bias, despite the application of rigorous selection criteria. Secondly, the findings are specific to ESCC patients receiving nICT, thereby limiting their generalizability to cases of adenocarcinoma or receipt of nCT or nCRT. To address this, a multi-center prospective study are planned to conduct to evaluate potential differences in treatment outcomes associated with various immunotherapeutic agents in esophageal adenocarcinoma. Thirdly, the sample size of patients receiving pembrolizumab was relatively small. Nonetheless, PSM was employed to mitigate baseline discrepancies. Future research should aim to increase the sample size and involve more medical centers to better capture prognostic differences. Besides, although our classification aligns with real-world clinical practice, we cannot entirely exclude potential heterogeneity among domestic agents. Prospective, large-scale head-to-head comparative studies are warranted to further clarify potential differences in treatment outcomes. Additionally, a limitation of this study was the lack of a detailed comparative analysis of toxicities and side effects, due to cross-departmental privacy policies.

## 5 Conclusion

This study demonstrated that domestic and imported immune checkpoint inhibitors have comparable efficacy and safety in patients with locally advanced ESCC receiving nICT. No significant differences were found in long-term survival outcomes or major postoperative complications. While imported drugs might provide a slight edge in terms of pathological response, both types are considered effective and viable for clinical application.

## Data Availability

The raw data supporting the conclusions of this article will be made available by the authors, without undue reservation.
